# Cilostazol protects hepatocytes against alcohol-induced apoptosis via activation of AMPK pathway

**DOI:** 10.1371/journal.pone.0211415

**Published:** 2019-01-29

**Authors:** Youn Ju Lee, Mi-Sun Shu, Jong-Yeon Kim, Yun-Hye Kim, Kyeong Hwa Sim, Woo Jung Sung, Jong Ryeol Eun

**Affiliations:** 1 Department of Pharmacology, School of Medicine, Catholic University of Daegu, Daegu, Korea; 2 Deparment of Physiology, School of Medicine, Yeungnam University, Daegu, Korea; 3 Department of Pathology, School of Medicine, Catholic University of Daegu, Daegu, Korea; 4 Department of Internal medicine, Myongj Hospital, Hanyang University College of Medicine, Goyang, Korea; Univerzitet u Beogradu, SERBIA

## Abstract

Alcoholic liver disease (ALD) is a worldwide health problem and hepatocyte apoptosis has been associated with the development/progression of ALD. However, no definite effective pharmacotherapy for ALD is currently available. Cilostazol, a selective type III phosphodiesterase inhibitor has been shown to protect hepatocytes from ethanol-induced apoptosis. In the present study, the underlying mechanisms for the protective effects of cilostazol were examined. Primary rat hepatocytes were treated with ethanol in the presence or absence of cilostazol. Cell viability and intracellular cAMP were measured. Apoptosis was detected by Hoechst staining, TUNEL assay, and caspase-3 activity assay. The roles of cAMP and AMP-activated protein kinase (AMPK) pathways in the action of CTZ were explored using pharmacological inhibitors and siRNAs. Liver from mice received ethanol (5 g/kg body weight) by oral gavage following cilostazol treatment intraperitoneally was obtained for measurement of apoptosis and activation of AMPK pathway. Cilostazol inhibited ethanol-induced hepatocyte apoptosis and potentiated the increases in cAMP level induced by forskolin. However, the anti-apoptotic effect of cilostazol was not reversed by an inhibitor of adenylyl cyclase. Interestingly, cilostazol activated AMPK and increased the level of LC3-II, a marker of autophagy. The inhibition of AMPK abolished the effects of cilostazol on LC3-II expression and apoptosis. Moreover, the inhibition of LKB1 and CaMKK2, upstream kinases of AMPK, dampened cilostazol-inhibited apoptosis as well as AMPK activation. In conclusion, cilostazol protected hepatocytes from apoptosis induced by ethanol mainly via AMPK pathway which is regulated by both LKB1 and CaMKK2. Our results suggest that cilostazol may have potential as a promising therapeutic drug for treatment of ALD.

## Introduction

Alcohol is an important risk factor for development of liver disease. Alcoholic liver disease (ALD) represents a spectrum of pathological conditions ranging from simple hepatic steatosis to alcoholic hepatitis, fibrosis and eventually to cirrhosis [1, 2]. Among cellular pathogenesis of ALD, hepatocyte apoptosis is a prominent feature of alcoholic hepatitis and hepatic fibrosis [3, 4]. The inhibition of hepatocellular apoptosis in various liver injury models has been shown to reduce liver damage and progression of liver diseases [5, 6]. Therefore, apoptosis has been considered as a target for therapeutic management of ALD.

Hepatocyte apoptosis by ethanol is mediated by various factors including ethanol metabolites, mitogen-activated protein kinases (MAPKs), reactive oxygen species (ROS) generation and TNFα production. It has been reported that cyclic AMP (cAMP) inhibits apoptotic process in hepatocytes via suppression of caspase activity and TNFα expression [7, 8]. Moreover, chronic ethanol exposure has shown to reduce hepatic cAMP in animal model which is associated with liver injury [9].

Cilostazol, a selective phosphodiesterase III (PDE III) inhibitor, has been widely used in clinical trials as an anti- platelet drug for the treatment of peripheral vascular diseases [10, 11]. In addition, cilostazol has shown protective effects in various liver injury models including hepatectomy [12], ischemia-reperfusion injury [13] and hepatic steatosis [14]. Very recently, it has been reported that cilostazol exerts protective effects on ethanol-induced hepatocyte damage through suppression of oxidative stress [15]. The pleiotropic effects of cilostazol have shown to be mediated by both cAMP-dependent and–independent pathways including antioxidant effect [16, 17] and AMP-activated protein kinase (AMPK) pathway [18, 19].

AMPK plays a critical role in controlling cellular energy homeostasis [20, 21]. In addition to its metabolic functions, AMPK plays a key role in regulation of cell survival/death. Recent study has shown that metformin protected liver from TNFα-induced apoptotic injury via AMPK-mediated caspase-3 inhibition [22], indicating anti-apoptotic role of AMPK. Moreover, the increased AMPK activity has been reported to alleviate various detrimental responses induced by ethanol in liver [23, 24]. Autophagy, a self-degradation of cellular components in lysosomes, has been reported to be an important homeostatic mechanism for liver function [25, 26]. Ethanol suppressed AMPK activation has been accompanied by downregulation of autophagic activity, where the increase in AMPK activation restored hepatic autophagy and subsequently reduced hepatic injury [27, 28]. These findings suggest possibility that the activation of AMPK might underlie the anti-apoptotic effects of cilostazol in liver exposed to ethanol. Indeed, in the present study, cilostazol suppressed hepatocyte apoptosis via AMPK pathway in *in vitro* and *in vivo* models.

## Materials and methods

### Animal care

Animals were purchased from the Samtako Inc. (Ohsan, Gyeonggi, Korea) and housed in a temperature (22 ± 2°C)- and humidity (55 ± 5%)- controlled room with a 12 h/12 h light/dark cycle (07:00–19:00 h). Animals were provided with standard laboratory chow and tab water *ad libitum*. After one week acclimatization, animals were used for experiments. A total of 35 rats and 50 mice were used in the experiments. Protocols involving animals were approved by Yeungnam University Animal Ethics Committee (approval No. YUMC-AEC2010-014).

### Isolation of hepatocytes

Male 7-week-old Sprague-Dawley rats were anesthetized with a cocktail of tiletamine HCl (25 mg/kg), zolazepam (25 mg/kg) and xylazine HCl (10 mg/kg). Then, hepatocytes were isolated using an *in situ* collagenase perfusion method as previously described [29]. Hepatocyte suspensions showed > 90% viability as determined by trypan blue exclusion. Cells were seeded on collagen-coated culture dishes in WME supplemented with 10% FBS, 100 U/ml penicillin, 100 μg/ml streptomycin, 2 mM L- glutamine, and 100 nM dexamethasone (10% WME). After 3 h, media were changed to WME containing 0.1% FBS, 100 U/ml penicillin, 100 μg/ml streptomycin, 2 mM L- glutamine, and 10 nM dexamethasone (0.1% WME).

### Ethanol and CTZ treatment *in vivo*

Seven-week old male C57BL/6 (18–22 g) mice were obtained from Central Lab. Animal Inc. (Seoul, Korea). After one week acclimatization, mice were received ethanol (5 g/kg body weight) by oral gavage and were sacrificed at different time (0 ~ 24 h). Ethanol was diluted with sterile water (32% w/v). For cilostazol treatment, mice were given an intraperitoneal injection of either vehicle (10% DMSO in PBS) or cilostazol (10 mg/kg/day) once a day for 4 days before ethanol administration. A single dose (5 g/kg body weight) of ethanol was given orally at one hour after the last treatment with cilostazol. Mice were sacrificed by anesthetization with a cocktail of tiletamine HCl (25 mg/kg), zolazepam (25 mg/kg) and xylazine HCl (10 mg/kg) and liver was collected 6 h after ethanol administration. The doses of cilostazol used in this study were selected based on previous reports by others [19].

### Measurement of cell viability

Cell viability was assessed by MTS assay using CellTiter 96 AQueous One Solution (Promega, Madison, WI, USA) containing 3-(4,5-dimethylthiazol-2-yl)-5-(3-carboxymethoxyphenyl)-2-(4-sulfophenyl)-2H-tetrazolium (MTS) and phenazine ethosulfate. Cells were seeded in collagen coated 96-well plates (2 x 10^4^ cells per well). After treatment of cells with ethanol, 20 μl of MTS solution was added to 100 μl of culture medium and the plates were incubated at 37°C for 4 h. The MTS tetrazolium compound is reduced to colored formazan product by metabolically active cells. Absorbance was measured at 490 nm.

### Hoechst staining

Nuclear morphology was detected by staining DNA using Hoechst 33342. Cells grown on collagen coated glass coverslips were fixed in ice cold methanol/acetic acid and then stained with Hoechst 33342 (5 μg/ml). After washing with deionized water, cells were mounted in 50% glycerol containing 20 mM citric acid and 50 mM disodium orthophosphate and observed under a fluorescent microscope (U-LH 100–3, Olympus).

### TUNEL assay

DNA fragmentation was detected with a terminal deoxynucleotidyl transferase (TdT)-mediated dUTP-nick end-labeling (TUNEL) assay kit (DeadEnd TM fluorometric TUNEL system; Promega, Madison, WI, USA) according to the manufacturer’s protocol. In brief, cells grown on collagen coated coverslips were fixed with a freshly prepared 4% paraformaldehyde for 25 min at 4°C. Then, cells were washed two times with ice-cold PBS and permeabilized with 0.2% Triton X-100 in PBS on ice for 5 min. The TdT and fluorescein-12-dUTP reactions were performed for 1 h at 37°C in a humidified box. TUNEL-positive cells were analyzed under a fluorescence microscope (U-LH 100–3, Olympus).

### Caspase-3 activity measurement

Caspase-3 activity was measured using a colorimetric assay kit according to the protocol provided by the manufacturer (R&D Systems, Inc., Minneapolis, MN, USA). Briefly, cells were lysed in lysis buffer (20 mM HEPES, 1 mM EDTA, 1 mM EGTA, 50 mM NaF, 10 mM β-glycerophosphate, 2 mM MgCl_2_, 150 mM NaCl, 10 mM KCl, 1% NP-40, 1 mM DTT, 1 mM benzamide, 1 mM PMSF, 10 μg/ml of aprotitin, leupeptin and pepstatin A). Then, proteins (150 μg) were incubated with caspase-3 substrate, Ac-DEVD-pNA-(7-amino-4 methyl coumarin) at 37°C for 1~2 h and the absorbance was measured at 405 nm. A recombinant caspase-3 enzyme (R&D Systems, Inc., Minneapolis, MN) was used for generating a standard and caspase-3 activity was calculated as ng/mg protein. The data were expressed as fold increase in activity.

### Western blotting

Equal amount of protein was separated by SDS-PAGE gel and transferred to nitrocellulose membrane. After blocking with 5% non-fat dry milk in Tris- buffered saline, the blots were incubated with the primary anti- phospho-AMPK (pAMPK, Cell Signaling, Beverly, MA, USA), anti-AMPK (Cell Signaling, Beverly, MA, USA), anti-phospho-acetyl-CoA carboxylase (pACC, Cell Signaling, Beverly, MA, USA), anti-ACC (Cell Signaling, Beverly, MA, USA), anti-phospho-LKB1 (pLKB1, Cell Signaling, Beverly, MA, USA), anti-LKB1 (Cell Signaling, Beverly, MA, USA), anti-phospho-CaMKK2 (pCaMKK2, Cell Signaling, Beverly, MA, USA), anti-phospho-mTOR (Cell Signaling, Beverly, MA, USA), anti-mTOR (Cell Signaling, Beverly, MA, USA), anti-LC3A/B (Cell Signaling, Beverly, MA, USA), anti-cleaved caspase-3 (C-caspase 3, Cell Signaling, Beverly, MA, USA), anti-cleaved PARP (C-PARP, Cell Signaling, Beverly, MA, USA), anti-GAPDH (Santa Cruz, Santa Cruz, CA, USA), and anti-CaMKK2 (Santa Cruz, Santa Cruz, CA, USA) antibodies bodies (1:1000 dilution into TBST/5% BSA) overnight at 4°C. The blots were incubated with secondary antibodies (1:3000 dilution into TBST/5% nonfat dry milk) conjugated to horseradish peroxidase. The protein bands were detected by the Super Signal (Pierce, Rockford, IL). The density of respective bands was analyzed by the Chemi-Doc XRS imaging system (Bio-Rad, Hercules, CA). The membranes were re-probed with anti-GAPDH antibody, which was used as loading control.

### Measurement of intracellular cAMP

The concentration of cAMP was measured using a cAMP EIA kit (Cayman, Ann Arbor, MI, USA) according to the manufacturer’s protocols. Briefly, cells were lysed in 1 ml of lysis buffer following exposure to indicated reagents and 50 μl of supernatant after centrifugation was used for assay and then absorbance was measured at 405 nm.

### Transfection with small-interfering RNA (siRNA)

Cells were transfected with 100 nM AMPK siRNA (50 nM α1(sc-270142) and 50 nM α2 (sc-155985), Santa Cruz Biotechnology, Santa Cruz, CA), LKB1 siRNA (#L-100539-02, Dharmacon, Lafayette, CO, USA), CaMKK2 siRNA (#L-097995-02, Dharmacon, Lafayette, CO, USA) or 100 nM scrambled siRNA (Ambion #AM4011, Invitrogen, Carlsbad, CA, USA) using lipofectamin RNAiMAX (Invitrogen, Carlsbad, CA) in OPTI-MEM (Invitrogen, Carlsbad, CA) according to the manufacturer’s protocol. After 24 h of transfection, culture medium was replaced with 0.1% WME. Cells were cultured for additional 24 h and then, harvested for further experiments.

### Histology and immunohistochemistry

Liver tissues were fixed in 4% paraformaldehyde. Paraffin-embedded tissue sections (4 μM thickness) were stained for hematoxylin and eosin (H&E). Protein expression level of cleaved caspase-3 was assessed by immunohistochemistry using Bond-III IHC stainer (Leica Biosystems, Nussloch, Germany) according to the manufacturer’s protocol. Liver sections were immunostained with a primary antibody against cleaved caspase-3 (1:200, Cell signaling, Beverly, MA, USA).

### Statistical analysis

Data are expressed as mean ± S.E.M. Statistical analyses were performed using the Student’s *t*-test for comparison of values between two groups or one way ANOVA followed by Tukey’s post hoc test for comparison of values among more than three groups. A value of *P* < 0.05 was considered significant. Values were analyzed using GraphPad Prism 4.0 software.

## Results

### Effects of cilostazol on ethanol-reduced viability of hepatocytes

**Fig 1:**
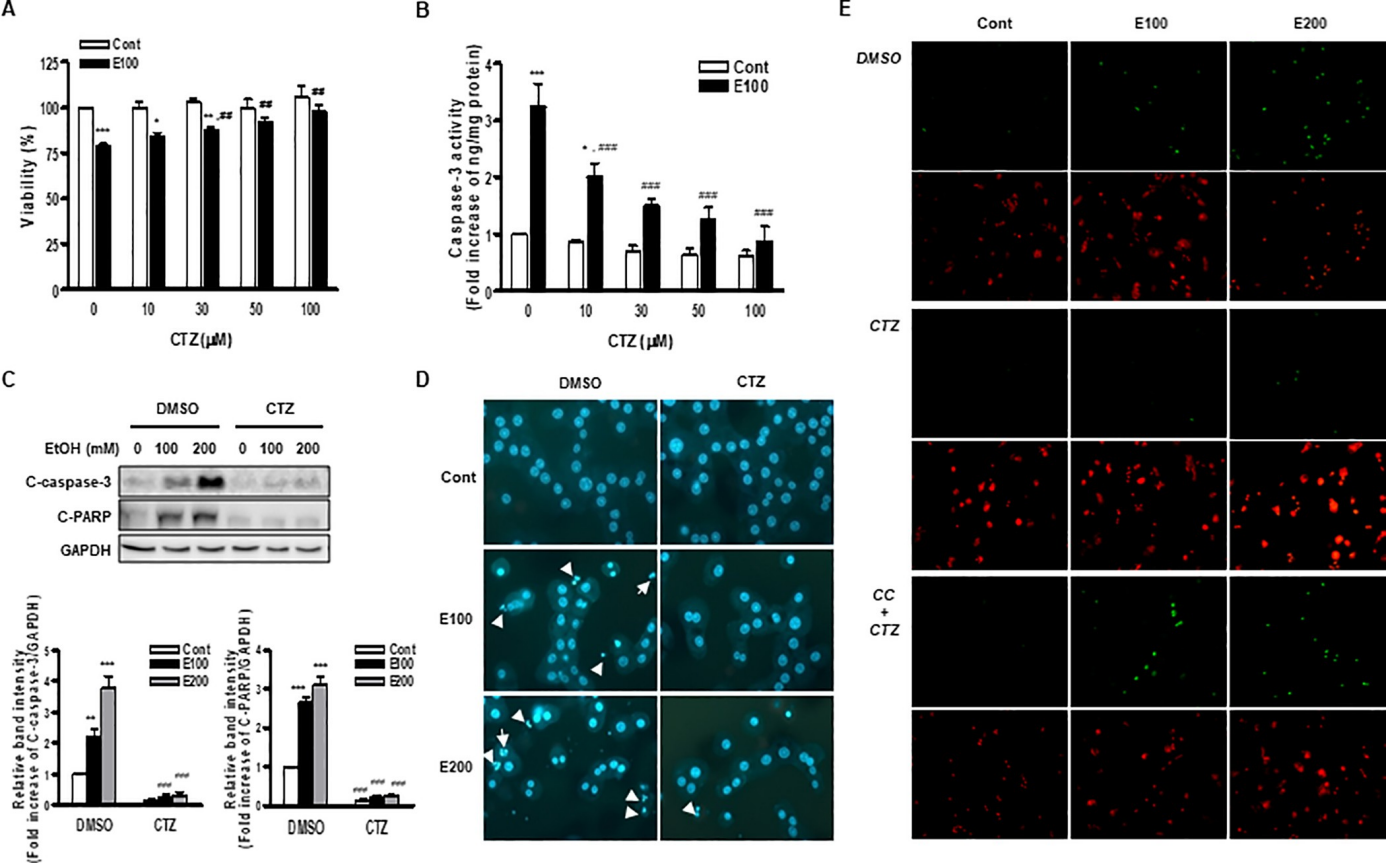
Effects of cilostazol on ethanol-induced apoptosis of hepatocytes. (A, B) Cells were treated with 100 mM of ethanol for 24 h in the presence or absence of different concentration of cilostazol (0 ~ 100 μM). Cell viability (A) and caspase-3 activity (B) were measured by MTS assay and activity assay as described in “methods”. (C—E) Cells were treated with 100 mM or 200 mM ethanol for 24 h in the presence or absence of cilostazol (100 μM) alone or together with compound C (10 μM). The cleaved caspase-3 (C-caspase-3) and cleaved PARP (C-PARP) were detected by Western blotting. The blots are representative of four independent experiments. Protein levels were normalized to GAPDH. The relative band intensities are presented (C). Nuclear morphology was monitored by fluorescence microscopy after staining cells with Hoechst 33342. Arrows indicate nuclear condensation or nuclear fragmentation (D). TUNEL positive cells were monitored by fluorescence microscopy after staining cells with propidium iodide (red) and TUNEL (green) (E). Representative microscopic images from three independent experiments were presented (magnification, x 400). Data represented as fold increases are mean±S.E.M. of three independent experiments. *P < 0.05, **P < 0.01 and ***P < 0.001 vs. control; ^##^*P* < 0.01 and ^###^*P* < 0.001 vs. corresponding DMSO treated cells. (Cont, control; EtOH, ethanol; E100, ethanol 100 mM; E200, ethanol 200 mM; CTZ, cilostazol; CC, compound C).

The protective effects of cilostazol on hepatocyte viability after ethanol exposure were determined. Treatment of cells with 100 mM of ethanol for 24 h reduced cell viability by about 20%. Pretreatment with cilostazol resulted in concentration-dependent improvement of cell viability and the effect was significant above 30 μM of cilostazol (Fig 1A).

### Effects of cilostazol on ethanol-induced apoptosis of hepatocytes

To determine the anti-apoptotic effect of cilostazol in hepatocytes, caspase-3 activity was assessed by activity assay and Western blotting. Ethanol significantly increased caspase-3 activity, which was suppressed by cilostazol in concentration-dependent manner (Fig 1B). The inhibitory effect of cilostazol was significant above 10 μM. Consistent result was observed by Western blotting. Ethanol increased the levels of cleaved caspase-3, an active form of caspase-3, and cleaved poly (ADP-ribose) polymerase (PARP), one of the main cleavage targets of casapse-3, which were almost completely reduced by cilostazol (Fig 1C).

Next, the apoptotic morphological changes were determined by Hoechst staining. Treatment with ethanol for 24 h increased the number of apoptotic cells with condensed chromatin and fragmented nuclei, which was reduced by pretreatment with cilostazol (Fig 1D). Similarly, ethanol (100 and 200 mM) increased the number of TUNEL-positive cells, which was almost completely prevented by cilostazol (Fig 1E).

### The roles of cAMP in the anti-apoptotic effects of cilostazol in hepatocytes

**Fig 2:**
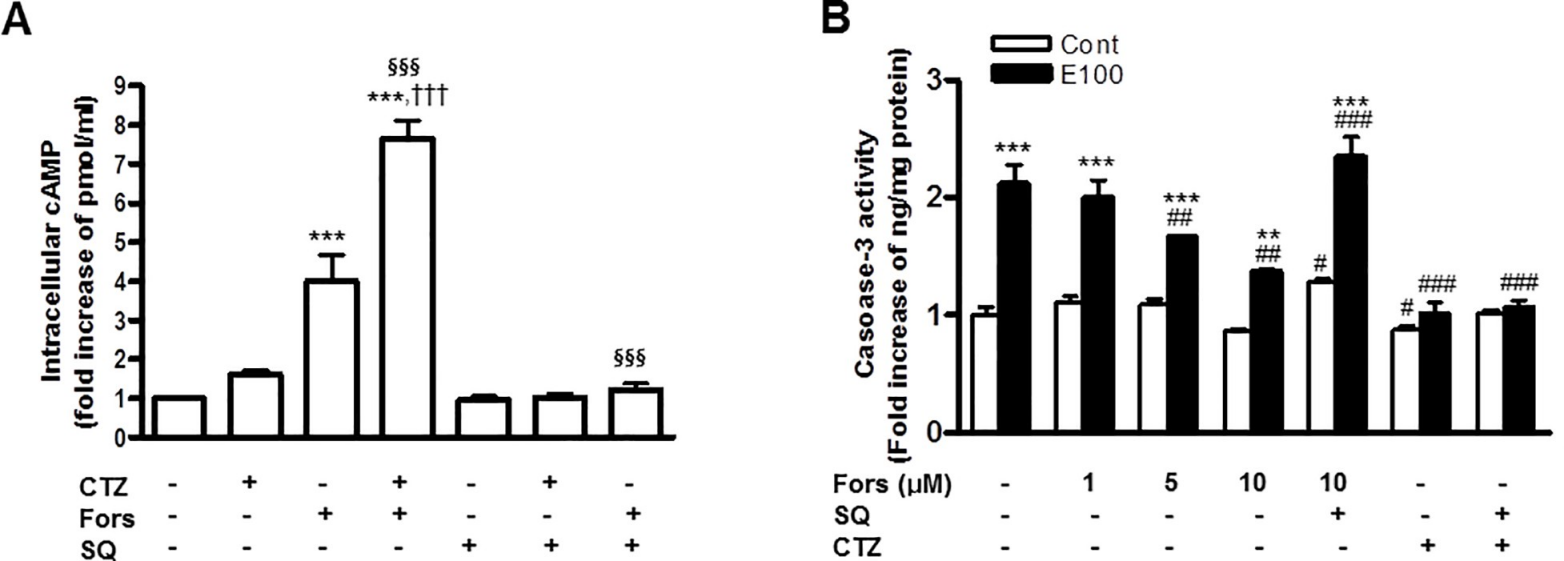
The role of cAMP in the effects of cilostazol on ethanol-induced apoptosis of hepatocytes. (A) Cells were treated with cilostazol (100 μM), forskolin (10 μM) for 30 min in the presence or absence of SQ22536 (400 μM). Intracellular cAMP concentration was measured. (B) Cells were treated with ethanol (100 mM) for 24 h in the presence of forskolin (1 ~ 10 μM), cilostazol (100 μM), or SQ22536 (400 μM). Caspase-3 activity was measured. Data represented as fold increases are mean±S.E.M. of three independent experiments. **P < 0.01 and ***P < 0.001 vs. control; ^#^*P* < 0.05, ^##^*P* < 0.01 and ^###^*P* < 0.001 vs. corresponding DMSO; ^†††^ *P* < 0.001 vs. corresponding cilostazol alone-treated cells, ^§§§^*P* < 0.001 vs. corresponding forskolin alone-treated cells. (Cont, control; E100, ethanol 100 mM; CTZ, cilostazol; Fors, forskolin; SQ, SQ22536).

Since many therapeutic effects of cilostazol are known to be attributed to the cAMP- dependent pathways, the role of cAMP in the anti-apoptotic effect of cilostazol was evaluated. Treatment with 10 μM of forskolin, an adenylyl cyclase activator, increased intracellular cAMP about four fold, which was almost completely blocked by SQ22536, an adenylyl cyclase inhibitor (Fig 2A). Although cilostazol alone slightly increased cAMP, cilostazol potentiated the cAMP elevation by forskolin (Fig 2A). Next, the role of cAMP in the inhibitory effect of cilostazol on the caspase-3 activity was measured. Treatment of cells with forskolin (1 ~ 10 μM) resulted in significant inhibition of ethanol-induced caspase-3 activity in concentration-dependent manner and the inhibitory effect of forskolin was reversed by SQ22536 (Fig 2B). However, SQ22536 failed to dampen the anti-apoptotic effect of cilostazol, indicating that the anti-apoptotic effect of cilostazol in hepatocytes is mainly mediated by a cAMP-independent pathway.

### The involvement of AMPK in the anti-apoptotic effects of cilostazol in hepatocytes

**Fig 3:**
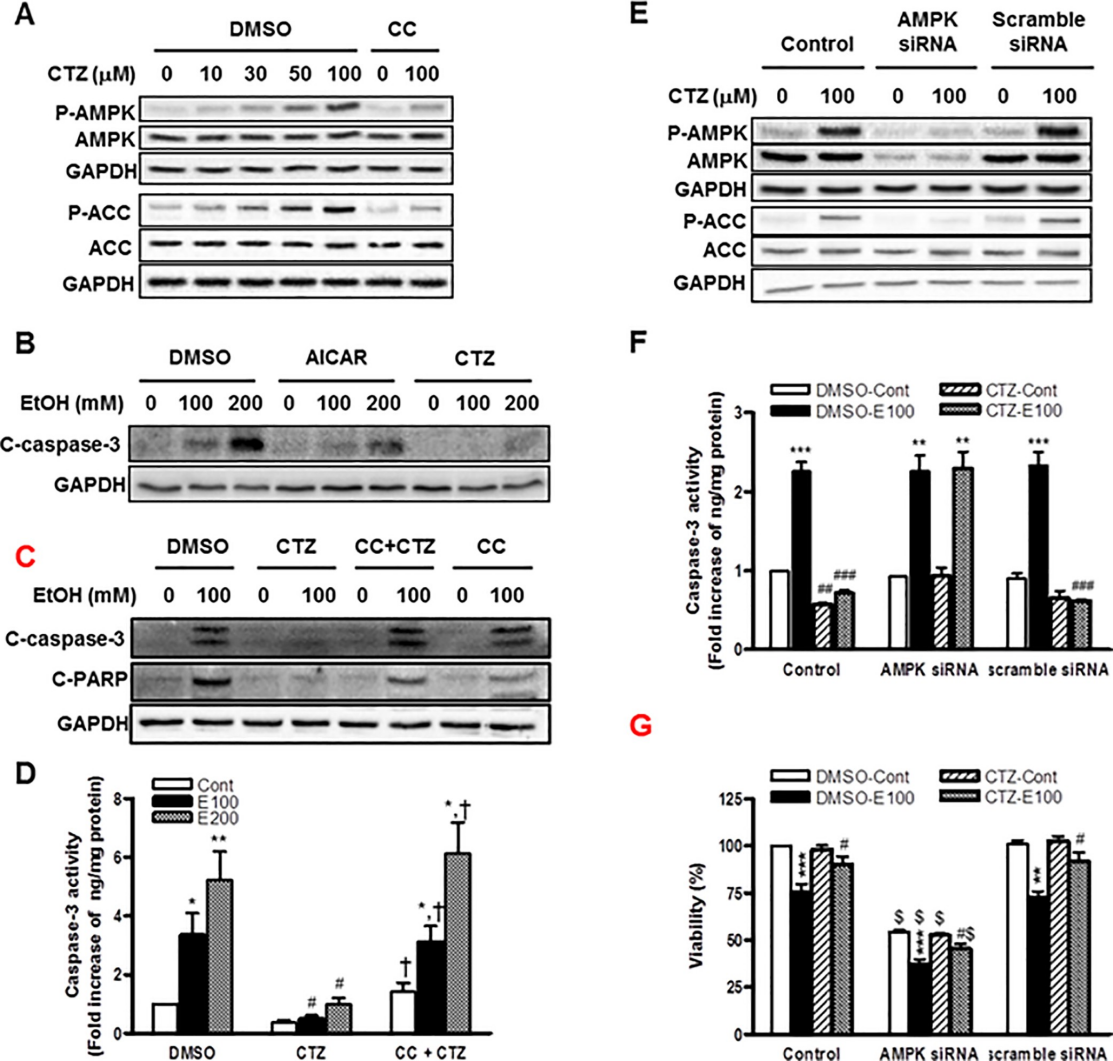
The role of AMPK in the effects of cilostazol on ethanol-induced apoptosis of hepatocytes. (A) Cells were treated with cilostazol (0 ~ 100 μM) in the presence or absence of compound C (10 μM) for 10 min. Phosphorylated- and total-AMPK and ACC were detected by Western blotting. (B—D) Cells were treated with ethanol (100 and 200 mM) in the presence of AICAR (100 μM), cilostazol (100 μM), compound C (10 μM) or compound C (10 μM) plus cilostazol (100 μM) for 24 h. Cleaved caspase-3 (C-caspase-3) or cleaved PARP (C-PARP) were detected by Western blotting (B, C). The caspase-3 activity was measured (D). (E) Cells were treated with AMPK siRNA or scramble siRNA for 48 h and then treated with cilostazol (100 μM) for 10 min. Phosphorylated- and total-AMPK and ACC were detected by Western blotting. (F, G) Cells were treated with AMPK siRNA or scramble siRNA for 24 h and then stimulated with ethanol (100 mM) in the presence or absence of cilostazol (100 μM) for 24 h. Caspase-3 activity (F) and cell viability (G) were measured. Representative microscopic images from three independent experiments were presented (magnification, x 400). The blots are representative of three independent experiments. Data represented as fold increases is mean±S.E.M. of three independent experiments. *P < 0.05, **P < 0.01 and ***P < 0.001 vs. control; ^#^*P* < 0.05, ^##^*P* < 0.01 and ^###^*P* < 0.001 vs. corresponding DMSO-treated cells; ^†^ *P* < 0.05 vs. corresponding cilostazol alone-treated cells; ^$^ *P* < 0.001 vs. corresponding scramble siRNA-treated cells. (Cont, control; E100, ethanol 100 mM; E200, ethanol 200 mM; CTZ, cilostazol; CC, compound C).

To determine whether the anti-apoptotic effects of cilostazol is mediated by AMPK pathway, the effect of cilostazol on AMPK signaling was examined. The activation of AMPK involves the phosphorylation of Thr 172 residue in α subunit of AMPK [30]. First, the activation of AMPK by cilostazol was examined by Western blotting using anti-phospho AMPKα Thr 172 antibody. Cilostazol increased phosphorylations of AMPK and acetyl-CoA carboxylase (ACC), a downstream target of AMPK, in a concentration-dependent manner. Phosphorylation of AMPK and ACC was effectively suppressed by compound C, an AMPK inhibitor (Fig 3A).

Next, the role of AMPK in ethanol-induced apoptosis was examined. AICAR, an AMPK activator, and cilostazol substantially suppressed the increased cleaved caspase-3 level (Fig 3B). The inhibitory effects of cilostazol on cleaved-caspase-3 and -PARP were reversed by co-treatment with compound C. Compound C itself had no effect on the basal- and ethanol-induced caspase-3 activation (Fig 3C). Consistent results were observed by caspase-3 activity assay (Fig 3D) and TUNEL assay (Fig 1E), where the anti-apoptotic effect of cilostazol was abolished by compound C. The involvement of AMPK in anti-apoptotic effect of cilostazol was further confirmed by using AMPK siRNA. As expected, AMPK siRNA significantly reduced AMPK activation and AMPK protein levels induced by cilostazol, whereas scramble siRNA had no effect (Fig 3E). Consequently, AMPK siRNA suppressed the phosphorylation of ACC without affecting ACC protein level (Fig 3E). Similar to the effect of compound C, AMPK siRNA abolished the inhibitory effect of cilostazol on the caspase-3 activity induced by ethanol (Fig 3F).These results suggest that the anti-apoptotic effect of cilostazol is mainly mediated by AMPK pathway. Consistently, AMPK siRNA reduced the protective effect of cilostazol on cell viability reduced by ethanol (Fig 3G). Interestingly, AMPK siRNA treatment decreased basal cell viability by almost 50%. This suggests that AMPK activation plays a pivotal role in hepatocyte survival mechanisms including anti-apoptotic process.

### The regulation of AMPK by LKB1 and CaMKK2 in cilostazol -treated hepatocytes

**Fig 4:**
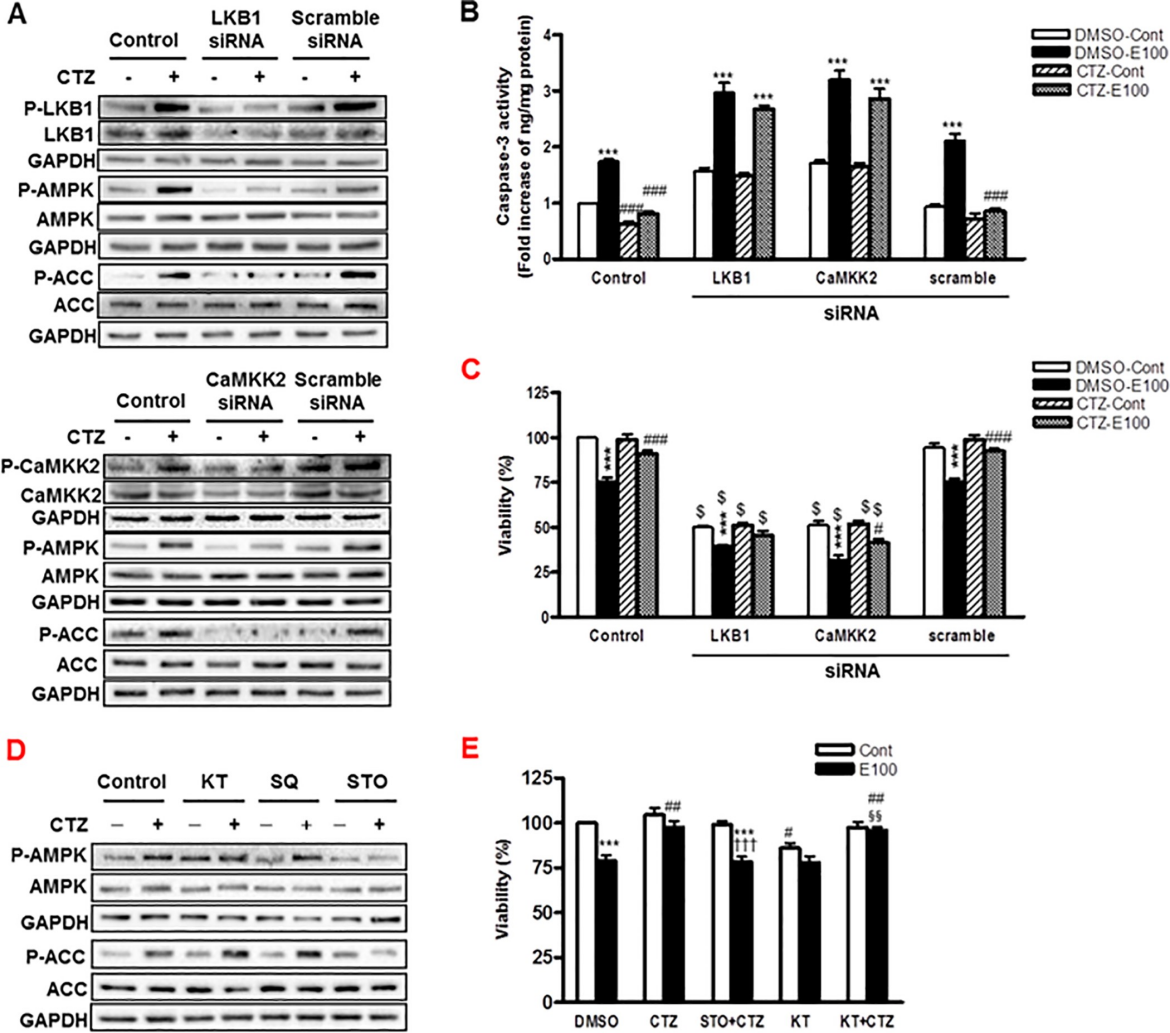
The regulation of AMPK by LKB1 and CaMKK2 in cilostazol treated hepatocytes. (A) Cells were treated with LKB1- and CaMKK2- siRNA or scramble siRNA for 48 h and then treated with cilostazol (100 μM) for 10 min. Phosphorylated- and total-LKB1, CaMKK2, AMPK and ACC were detected by Western blotting. (B, C) Cells were treated with LKB1- and CaMKK2- siRNA or scramble siRNA for 24 h and then stimulated with ethanol (100 mM) in the presence or absence of cilostazol (100 μM) for 24 h. Caspase-3 activity (B) and cell viability (C) were measured. (D) Cells were treated with cilostazol (100 μM) for 10 min in the presence or absence of KT5720 (1 μM), SQ22536 (400 μM) or STO-609 (5 μM). Phosphorylated- and total- AMPK and ACC were detected by Western blotting. (E) Cells were treated with ethanol (100 mM) in the presence or absence of cilostazol (100 μM), STO-609 (5 μM) or KT5720 (1 μM) for 24 h. Then, cell viability was measured by MTS assay. The blots are representative of three independent experiments. Data represented as fold increases is mean±S.E.M. of three independent experiments. ***P < 0.001 vs. control; ^#^*P* < 0.05, ^##^*P* < 0.01 and ^###^*P* < 0.001 vs. corresponding DMSO-treated cells; ^$^ *P* < 0.001 vs. corresponding scramble siRNA-treated cells; ^†††^*P* < 0.001 vs. corresponding cilostazol alone-treated cells; ^§§^*P* < 0.01 vs. corresponding KT5720 alone-treated cells. (Cont, control; E100, ethanol 100 mM; CTZ, cilostazol; KT, KT5720; SQ, SQ22536; STO, STO-609).

AMPK activation can be regulated by various upstream kinases and cAMP/PKA-dependent pathway. Since liver kinase B1 (LKB1) and calcium/calmodulin-dependent protein kinase 2 (CaMKK2) are well known upstream kinases responsible for the phosphorylation of α subunit of AMPK [31], their involvement in AMPK activation by cilostazol was examined. Cilostazol increased the phosphorylation of both LKB1 and CaMKK2 (Fig 4A). Both LKB1- and CaMKK2- siRNA reduced phosphorylation of AMPK and ACC induced by cilostazol (Fig 4A), suggesting that both LKB1 and CaMKK2 are involved in cilostazol increased AMPK activation. In addition, both LKB1- and CaMKK2- siRNA abolished the inhibitory effect of cilostazol on the caspase3 activity increased by ethanol (Fig 4B). Similar to AMPK siRNA, LKB1- and CaMKK2-specific siRNA significantly reduced both basal and cilostazol increased cell viability (Fig 4C).

The upstream regulators of AMPK were further confirmed with chemical inhibitors. STO-609, a CaMKK2 inhibitor decreased the phosphorylation of AMPK and ACC induced by cilostazol. However, KT5720, an inhibitor of cAMP-dependent protein kinase A, and SQ22536 had no effect on cilostazol-increased AMPK phosphorylation (Fig 4D). Consistently, the increased cell viability by cilostazol was revered by STO-609, but not by KT5720 (Fig 4E). These results indicate that cilostazol increased AMPK activation via LKB1- and CaMKK2-dependent pathways.

### The increase in AMPK/mTOR-dependent autophagy by cilostazol

**Fig 5:**
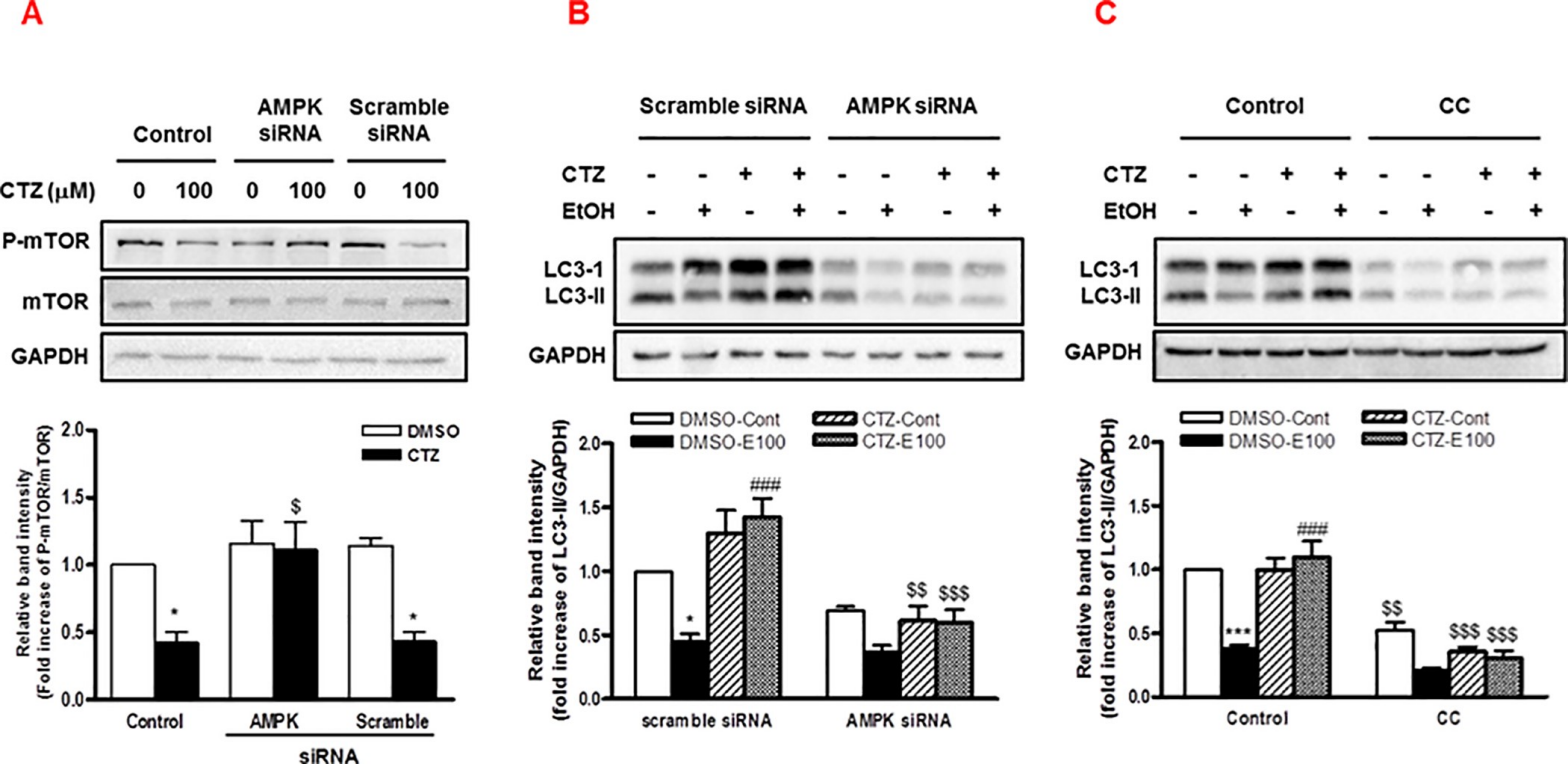
The effect of cilostazol on ethanol reduced autophagy in hepatocytes. **(**A) Cells were treated with AMPK siRNA or scramble siRNA for 48 h and then treated with cilostazol (100 μM) for 10 min. Phosphorylated- and total-mTOR were detected by Western blotting. (B, C) Cells were treated with AMPK siRNA, scramble siRNA or compound C (10 μM) and then stimulated with ethanol (100 mM) in the presence or absence of cilostazol (100 μM) for 24 h. LC3 level was detected by Western blotting. The relative band intensities are presented. Data represented as fold increases is mean±S.E.M. of three independent experiments. *P < 0.05 and ***P < 0.001 vs. vehicle control; ^###^*P* < 0.001 vs. corresponding DMSO-treated cells; ^$^*P* < 0.05, ^$$^*P* < 0.01 and ^$$$^*P* < 0.001 vs. corresponding scramble siRNA-treated cells. (Cont, control; E100, ethanol 100 mM; CTZ, cilostazol; CC, compound C).

The dysregulation of autophagic function has been suggested to be an important hepatic pathology induced by ethanol [27, 28, 32, 33]. Recently, it has been reported that cilostazol promoted autophagy via LKB1/AMPK/mTOR pathway, thereby increasing the degradation of β-amyloid in neuronal cells [34, 35]. AMPK activation has shown to induce autophagy by suppressing mammalian target of rapamycin (mTOR) signaling [36, 37]. Therefore, we examined whether the activation of AMPK signaling by cilostazol affects autophagy function in hepatocytes exposed to ethanol. Cilostazol significantly decreased the phosphorylation of mTOR and this inhibitory effect was abolished by AMPK siRNA (Fig 5A). As reported by others [27, 28, 33], ethanol reduced the level of Light Chain 3 (LC3)-II, a marker of autophagy, which was recovered by cilostazol in control cells treated with scramble siRNA. However, AMPK siRNA significantly reduced LC3-II level increased by cilostazol (Fig 5B). Consistently, compound C significantly reduced cilostazol effect on LC3-II expression (Fig 5C). These results suggest that cilostazol increased autophagy in hepatocytes via AMPK/mTOR dependent pathway.

### The effects of cilostazol on liver cell apoptosis in acute ethanol treated mice

**Fig 6:**
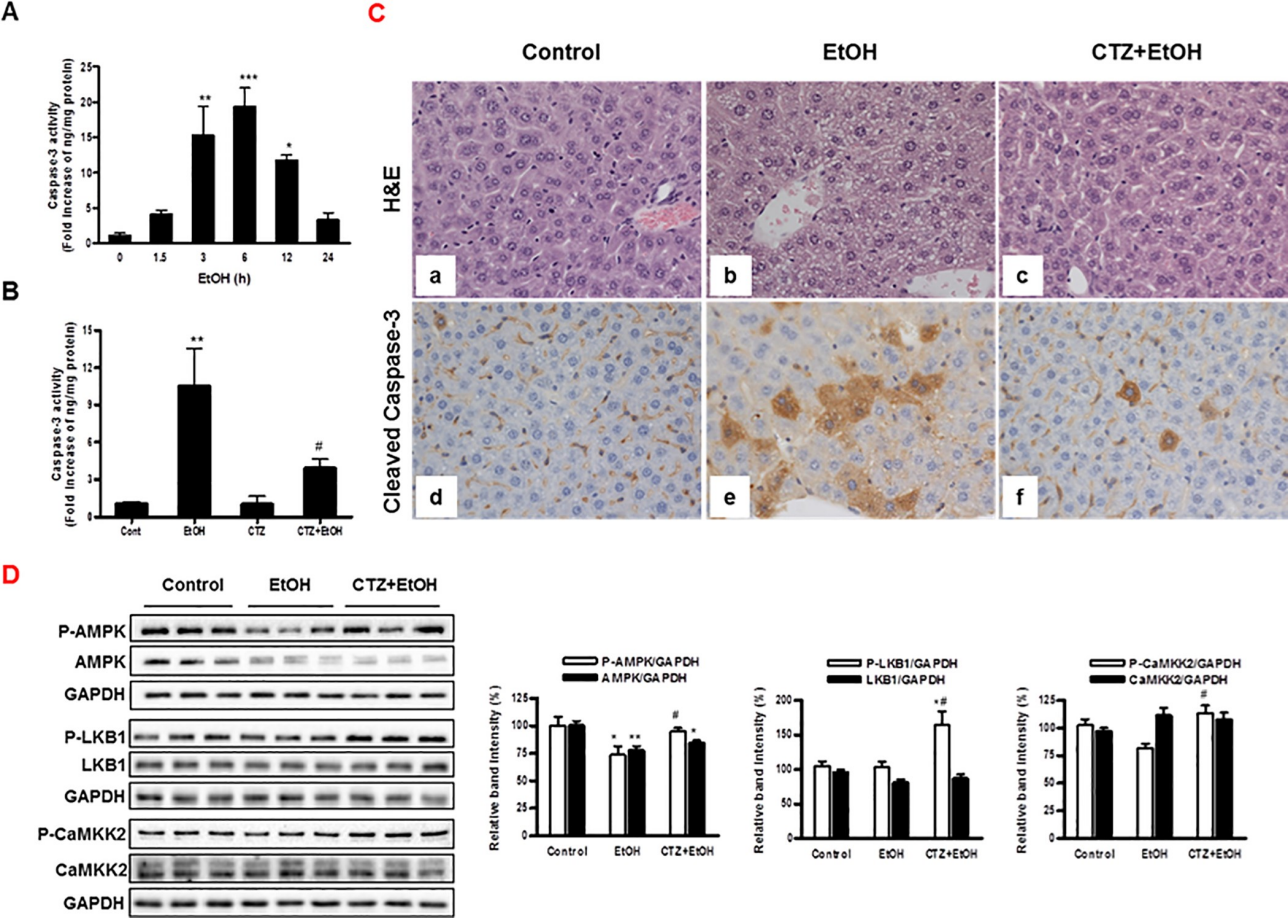
Effects of cilostazol on apoptosis and AMPK signaling in liver from binge alcohol treated mice. (A) After ethanol binge drinking (5 g/kg body weight) by oral gavage, caspase-3 activity was measured at different times. (B—D) Mice were subjected to intraperitoneal injection of cilostazol (10 mg/kg/day) for 4 days following a single dose of ethanol (5 g/kg) intake. Liver samples were taken at 6 h after ethanol intake. (C) Liver sections were stained with H&E (a-c) or anti-cleaved caspase-3 antibody (d-f). Cleaved caspase-3 positive cells showed cytoplasmic tan staining (magnification, x 400). Caspase-3 activity and AMPK signaling were measured by activity assay (B) and Western blotting (D), respectively. Three representative lanes of five different samples are shown in each group. Data represented as fold increases (A, B) or % of relative band intensity (D) are mean±S.E.M. (n = 5 mice per group). *P < 0.05, **P < 0.01, ***P < 0.001 vs. corresponding control; ^#^*P* < 0.05 vs. corresponding ethanol alone-treated group. (Cont, control; EtOH, ethanol; CTZ, cilostazol).

To validate the physiological relevance of anti-apoptotic effects of cilostazol, caspase-3 activity in liver from acute ethanol (5 g/kg body weight) treated mice was measured by activity assay and immunohistochemistry. Ethanol treatment significantly increased caspase-3 activity after 3 h of ethanol feeding with maximum increase at 6 h (Fig 6A), which was reduced by pretreatment with cilostazol (10 mg/kg of body weight) (Fig 6B). Histologically, microsteatosis of hepatocytes was frequently identified in ethanol group (Fig 6Cb), which was improved in cilostazol treated group (Fig 6Cc). Sinusoidal or periportal hepatitis was not revealed in all three groups under these experimental conditions, but a significant increase in the expression of cleaved caspase-3 was observed in the cytoplasm of hepatocytes from ethanol-fed mice as compared to control mice (Fig 6Cd and 6Ce). The level of cleaved caspase-3 expression was substantially reduced in cilostazol treated group (Fig 6Cf). The involvement of AMPK signaling in the action of cilostazol in liver was determined by Western blotting. In consistent with previous reports showing the decreased AMPK phosphorylation by binge or chronic ethanol feeding [38], AMPK phosphorylation was decreased by ethanol feeding, which was recovered by cilostazol (Fig 6D). Interestingly, the protein level of AMPK was also reduced by ethanol. However, cilostazol had no effect on AMPK protein level. Although the levels of LKB1 and CaMKK2 phosphorylation were not affected by ethanol feeding, cilostazol significantly increased LKB1 and CaMKK2 phosphorylation compared to ethanol feeding (Fig 6D). These suggest that cilostazol may protect liver from ethanol induced steatosis and apoptosis, two most important causal factors for alcoholic liver injury, by recovering AMPK signaling through LKB1 and CaMKK2 activation.

## Discussion

AMPK has recently emerged as an important regulator of various liver functions. Therefore, AMPK has been suggested to be a crucial therapeutic target to treat liver injury including steatosis, inflammation and apoptosis [39]. Cilostazol has been reported to have protective effects on diverse disease models including liver damage. The beneficial effects of cilostazol are mediated by multiple signaling pathways. However, the involvement of AMPK in the effects of cilostazol on hepatocyte apoptosis induced by ethanol has not been determined. In the present study, we have shown that cilostazol suppressed ethanol-induced hepatocyte apoptosis in *in vitro* primary hepatocyte and *in vivo* alcohol drinking mice. The pharmacological and gene knockdown approaches clearly demonstrated that the anti-apoptotic effect of cilostazol was mediated mainly through AMPK-dependent pathway. Of interest, the protective effect of cilostazol was cAMP/PKA independent although cAMP has shown an anti-apoptotic role. The activation of AMPK by cilostazol was down regulated by both LKB1- and CaMKK2- siRNA, which abrogated the anti-apoptotic effect of cilostazol. In addition, pharmacological inhibitor of CaMKK2, STO-609, but not inhibitors of cAMP/PKA pathway blunts AMPK activation, supporting the anti-apoptotic role of cilostazol via LKB1-CaMKK2/AMPK pathway. The protective effect of cilostazol on hepatocyte apoptosis is consistent with a recent report, in which cilostazol prevented hepatocyte apoptosis induced by ethanol by ameliorating ROS generation [15].

The increasing evidence suggests that the impairment of AMPK activity is associated with various liver diseases such as metabolic disorders, inflammation, and cancer. Ethanol treatment both *in vitro* and *in vivo* has shown to decrease hepatic AMPK activation [40], which was associated with development of steatosis, inflammation and apoptosis. Therefore, the identification of a reagent activating AMPK signaling can provide a good strategy to treat ALD. Consistently, our present study shows that the suppressive effect of cilostazol on hepatocyte apoptosis was abolished by AMPK inhibitor, compound C, and gene silencing of AMPKα, indicating that the anti-apoptotic effect of cilostazol is mediated by AMPK-dependent pathway.

The activation of AMPK composed of catalytic α subunit and regulatory β and γ subunits is regulated by several independent mechanisms. It is generally known that AMPK activity is regulated by intracellular energy status. In response to energy depletion, the increased AMP/ATP ratio increased AMPK activation via binding of AMP to AMPKγ subunit which leads to its conformational change [41]. The best known as an AMPK activator, AICAR is converted to ZMP within cells and then activates AMPK via mechanism mimicking AMP [42]. Similarly, metformin and thiazolidinediones (TZDs), which are used to treat diabetes in clinical situation, have shown to increase AMP/ATP ratio by inhibiting Complex I of the respiratory chain and subsequently activate AMPK [43]. However, metformin has also shown to activate AMPK without altering AMP/APT ratio [44]. It has been reported that ethanol treatment reduced ATP level in hepatocyte which was recovered by cilostazol [15]. This suggests that cilostazol may activate AMPK via AMP-independent pathway. The activation of AMPK is increased by upstream kinases including LKB1 and CaMKK2. Depending on cell types and stimuli, AMPK is phosphorylated by specific upstream kinases. In the present study, AMPK phosphorylation induced by cilostazol was abolished by both LKB1- and CaMKK2-siRNA. Although LKB1 is mainly involved in AMPK activation, CaMKK2 also acts as an upstream kinase of AMPK in response to the increased intracellular calcium [31]. Interestingly, cilostazol has shown to increase intracellular calcium in endothelial cells [45]. However, the mechanisms responsible for cilostazol regulated LKB1 and CaMKK2 in hepatocytes are not completely understood and further investigations are needed in the future study.

In contrast to our finding, the increase of intracellular cAMP underlies the various pharmacological effects of cilostazol. Moreover, cilostazol induced AMPK phosphorylation in neuronal cells was abolished by KT5720, a PKA inhibitor [35]. Intracellular cAMP is produced by activation of adenylyl cyclase and degraded by PDE. cAMP, a second messenger molecule, is known to be involved in a wide range of cellular responses including cell proliferation, differentiation, inflammation, and apoptosis. In liver, the involvement of the cAMP signaling pathway in both cell survival and apoptosis depending on stimuli has been demonstrated. It has been reported that chronic ethanol caused a decrease of cAMP and activation of adenylyl cyclase prevented ethanol-induced liver injury [8, 46], indicating the protective role of cAMP. In agreement with these reports, our results show that forskolin significantly increased intracellular cAMP level and suppressed caspase-3 activation increased by ethanol (Fig 2A and 2B). Although cilostazol alone marginally increased cAMP level under this condition, CTZ potentiated forskolin effect on cAMP elevation. However, adenylyl cyclase inhibitor SQ22536 failed to abolish the anti-apoptotic effect of cilostazol. Furthermore, neither SQ22536 nor KT5720 had effect on cilostazol-induced AMPK activation. These suggest that the protective effect of cilostazol on ethanol-induced apoptosis is mainly mediated by cAMP-independent pathways. Similarly, previous reports have shown that cilostazol did not affect intracellular cAMP level and elicited its beneficial effects via cAMP-independent pathways [47, 48].

Autophagy is considered as an important cellular mechanism for the maintenance of liver functions by lysosomal degradation and recycling of cellular components [25, 26], where AMPK activation plays a critical role in autophagy induction by inhibiting mTOR signaling [36, 37]. Acute or chronic ethanol treatment induced a decrease in hepatic autophagy, and restoration of the autophagic activity ameliorated the resulting pathogenesis including steatosis, oxidative stress, and apoptosis [27, 28, 32, 33]. In the present study, cilostazol decreased mTOR phosphorylation and restored the level of LC3-II expression decreased by ethanol, which was AMPK dependent. This suggests that cilostazol improved autophagy function in hepatocytes via AMPK/mTOR dependent pathway, which may be responsible in part for the protective effect of cilostazol from hepatic fat accumulation and apoptosis induced by ethanol.

Oxidative stress is one of important contributing factors for ethanol-induced liver injury and numerous anti-oxidants have shown beneficial effects on ALD. We have also overserved that trolox, an antioxidant, increased hepatocyte viability reduced by ethanol (S1A Fig). In recent study, cilostazol prevented ethanol-induced hepatocyte apoptosis via ameliorating ROS generation [15]. Consistently, we have also observed that cilostazol completely blocked ROS generation induced by ethanol in hepatocytes (S1B Fig). However, this anti-oxidant effect of cilostazol was not reversed by inhibition of either cAMP/PKA pathway or AMPK pathway (S1B Fig). Therefore, cilostazol may protect liver cells from ethanol via AMPK-dependent and–independent pathways including antioxidant effect.

Taken together, cilostazol protected hepatocytes from apoptosis induced by ethanol and its effect is mainly mediated by activation of AMPK pathway and dampening ROS generation. The activation of AMPK is regulated by upstream kinases LKB1 and CaMKK2. Considering that cilostazol has been safely used in various clinical situations for the treatment of peripheral vascular diseases, cilostazol may be a promising a novel pharmacological therapy for ALD.

## Supporting information

S1 Fig The effects of cilostazol on ROS generation by ethanol. (A) Cells were treated with ethanol (100 mM) for 24 h in the presence or absence of trolox (100 μM). Cell viability was measured by MTS assay. (B) Cells were treated with ethanol (100 mM) for 24 h in the presence or absence of trolox (100 μM), cilostazol (50 and 100 μM) alone or together with compound C (10 μM), STO-609 (5 μM), KT5720 (1 μM) or SQ22536 (400 μM). ROS accumulation was determined by measuring DCF fluorescence. Data represented as fold increase of control are mean±S.E.M. of three independent experiments. ***P < 0.001 vs. control; ^#^*P* < 0.05, ^##^*P* < 0.01 and ^###^*P* < 0.001 vs. corresponding DMSO-treated cells. (Cont, control; E100, ethanol 100 mM; CTZ, cilostazol; CC, compound C; STO, STO-609; KT, KT5720; SQ, SQ22536).
(TIFF)

S2 Fig Uncropped scans of blots. (DOCX)
