# Supplementary figures and images for: Cilostazol protects hepatocytes against alcohol-induced apoptosis via activation of AMPK pathway
Source: PLoS One. 2019 Jan 29;14(1):e0211415. doi: 10.1371/journal.pone.0211415 (PMC6350983; doi:10.1371/journal.pone.0211415)

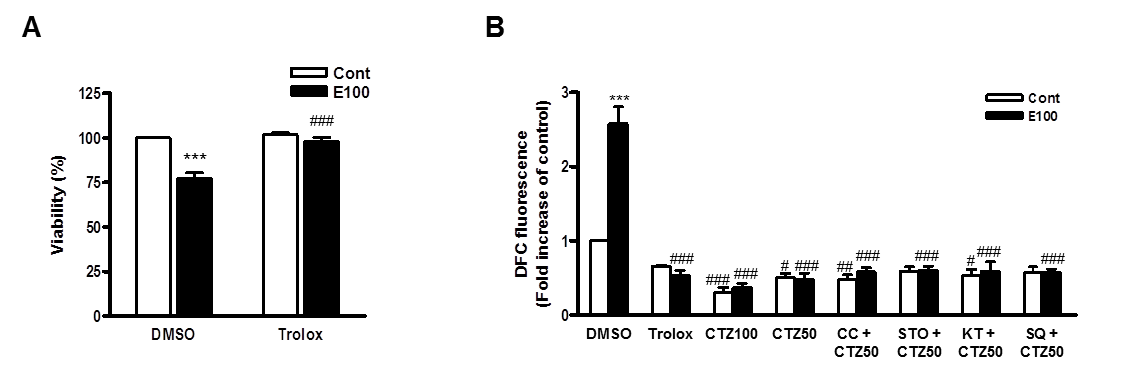

Supplement: S1 Fig — (A) Cells were treated with ethanol (100 mM) for 24 h in the presence or absence of trolox (100 μM). Cell viability was measured by MTS assay. (B) Cells were treated with ethanol (100 mM) for 24 h in the presence or absence of trolox (100 μM), cilostazol (50 and 100 μM) alone or together with compound C (10 μM), STO-609 (5 μM), KT5720 (1 μM) or SQ22536 (400 μM). ROS accumulation was determined by measuring DCF fluorescence. Data represented as fold increase of control are mean±S.E.M. of three independent experiments. ***P < 0.001 vs. control; #P < 0.05, ##P < 0.01 and ###P < 0.001 vs. corresponding DMSO-treated cells. (Cont, control; E100, ethanol 100 mM; CTZ, cilostazol; CC, compound C; STO, STO-609; KT, KT5720; SQ, SQ22536). (TIFF) [file pone.0211415.s001.tiff]

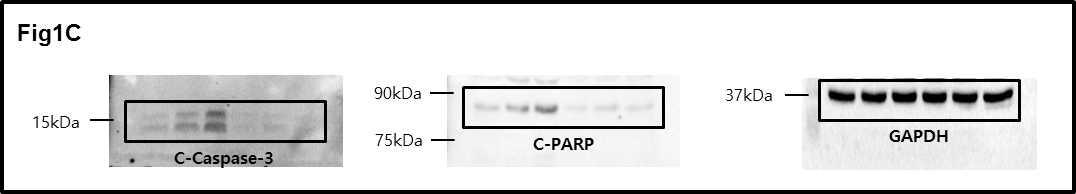

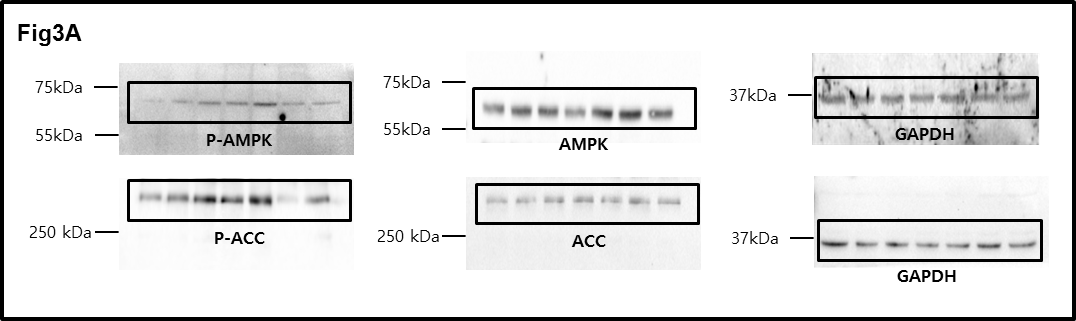

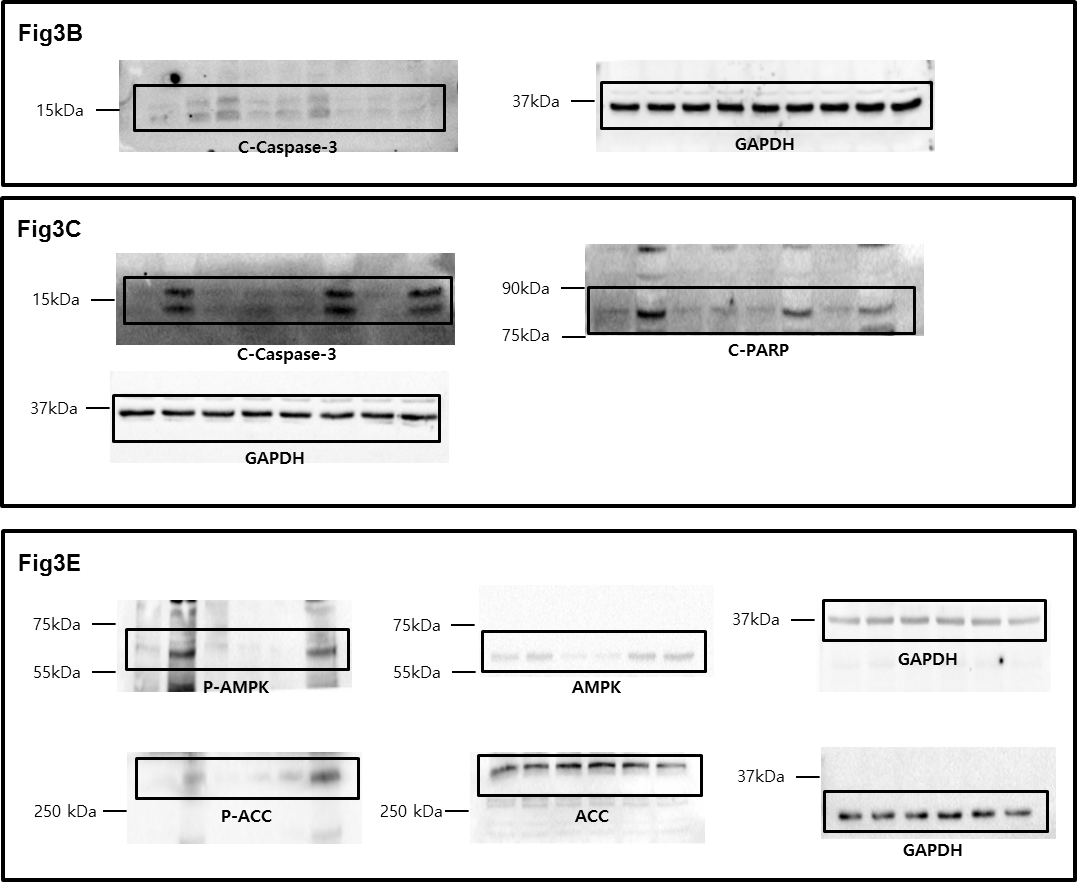


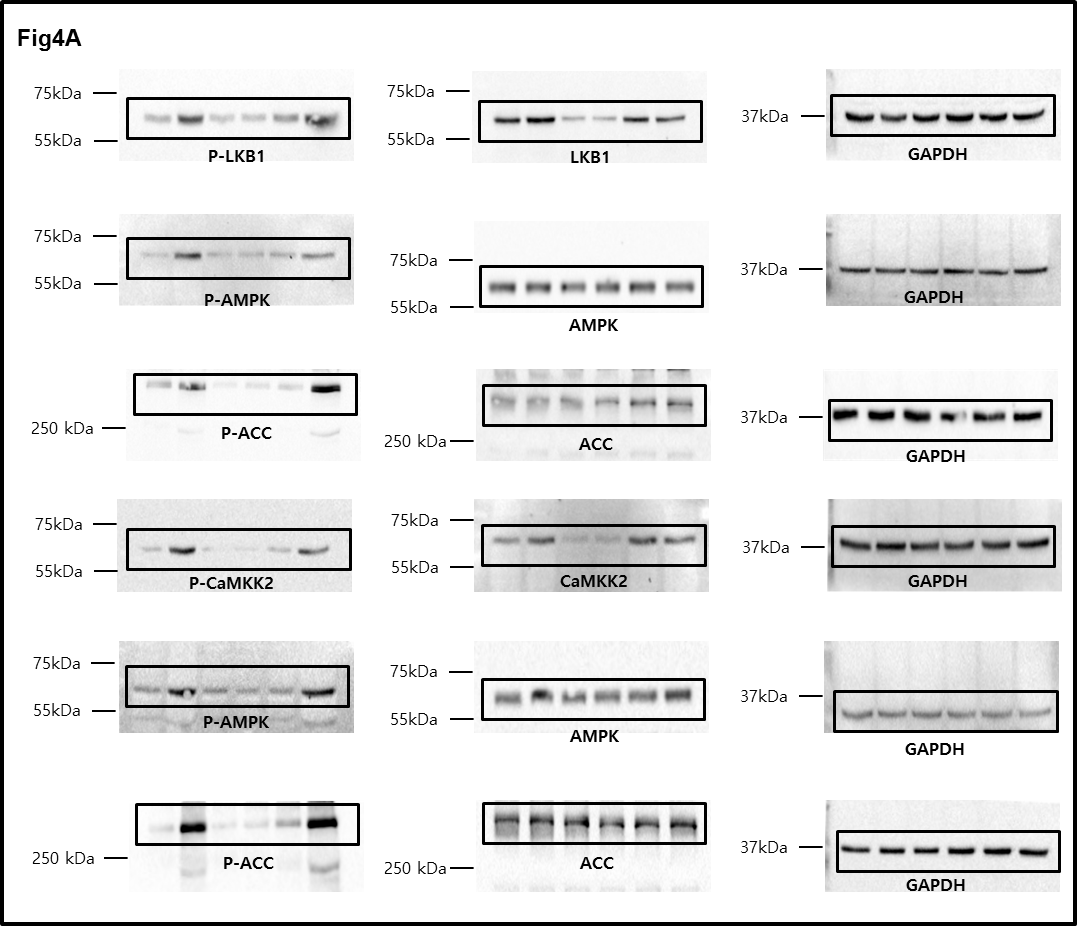


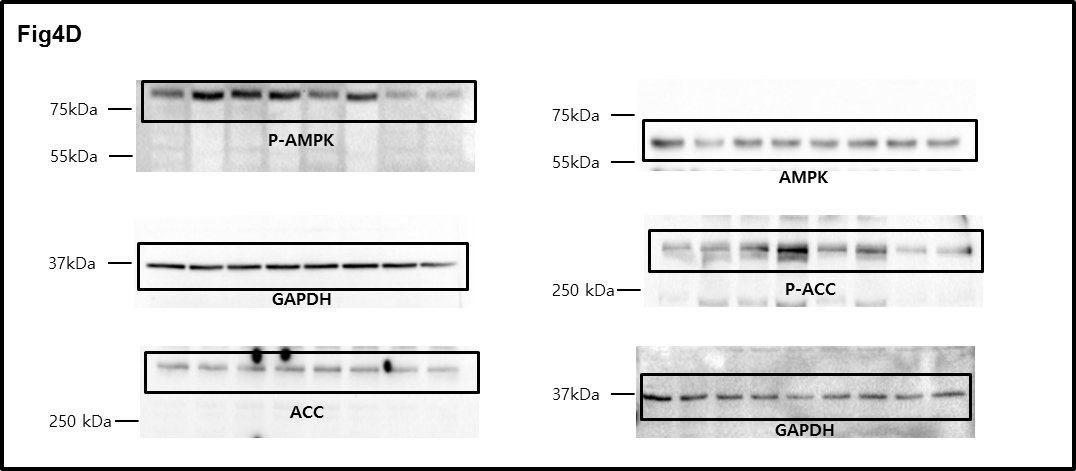


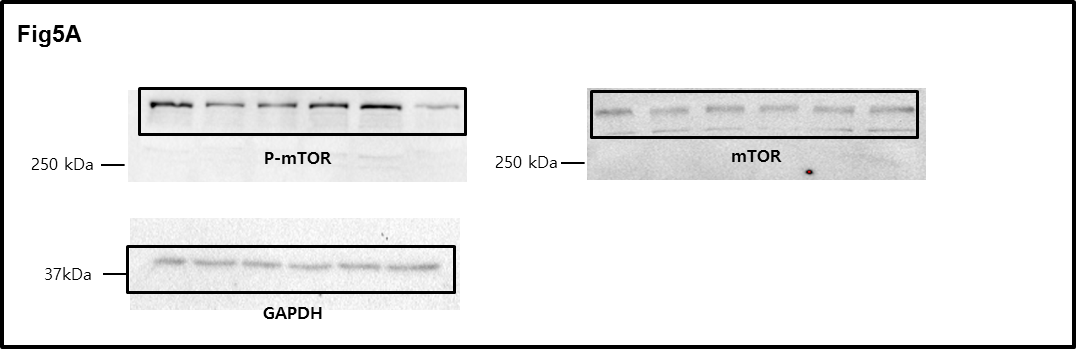


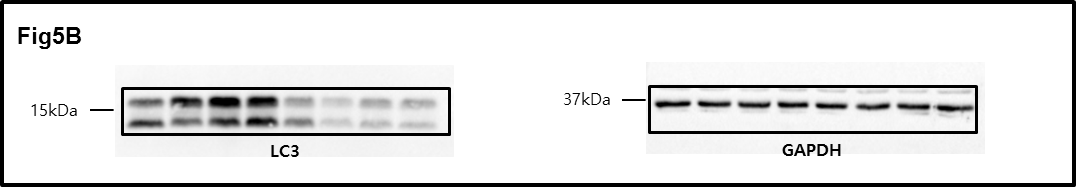


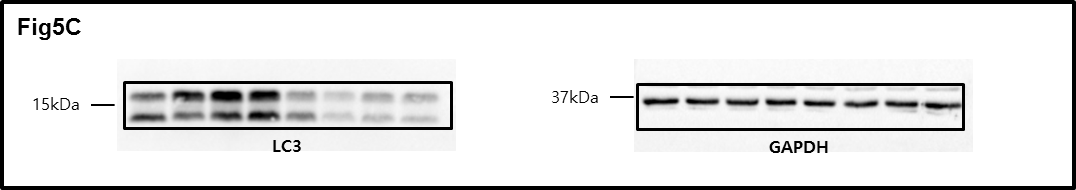


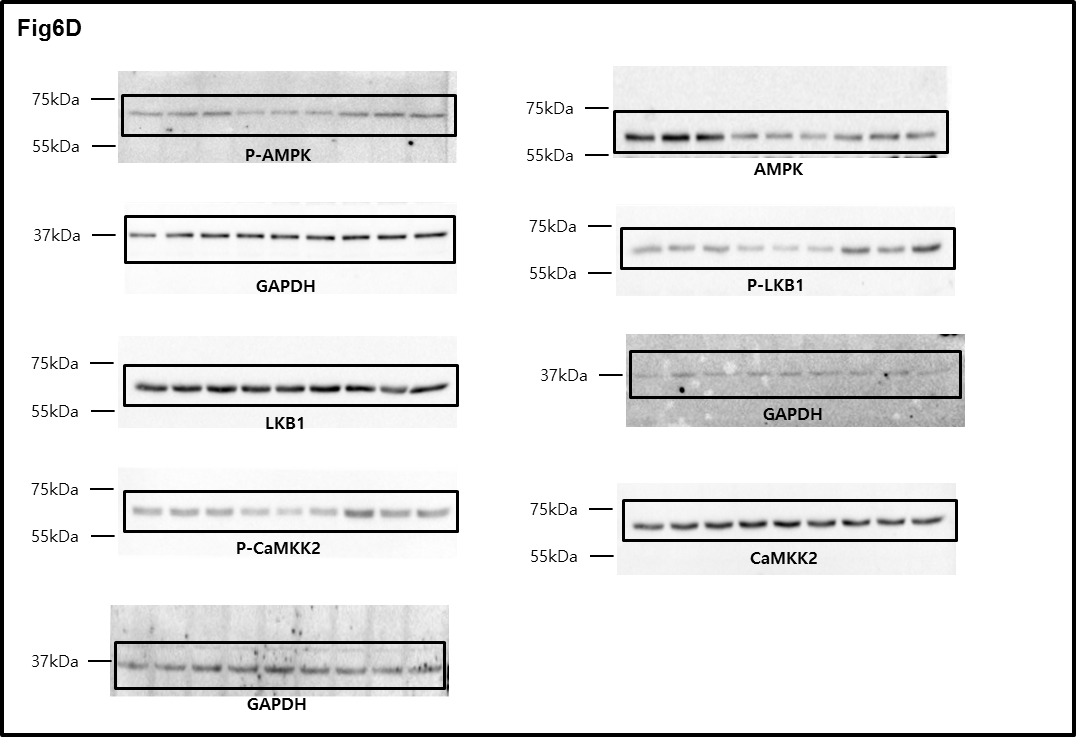

Supplement: S2 Fig — (DOCX) [file pone.0211415.s002.docx]
